# Socioeconomic inequality in the completion of the continuum of care for maternal, newborn, and child health in Malawi

**DOI:** 10.3389/frph.2026.1919232

**Published:** 2026-09-30

**Authors:** Redson Mwandama, Spy Munthali

**Affiliations:** Department of Economics, School of Law, Economics and Government, University of Malawi, Zomba, Malawi

**Keywords:** concentration index, continuum of care, decomposition, inequality of opportunity, Malawi, maternal health, socioeconomic inequality

## Abstract

**Background:**

Despite improvements in maternal, newborn, and child health (MNCH) service coverage in Malawi, completion of the continuum of care (CoC) remains low and unequally distributed across socioeconomic groups. This study quantified socioeconomic inequality in MNCH CoC completion and its determinants.

**Methods:**

We analyzed pooled data from the 2010, 2015–16, and 2024 Malawi Demographic and Health Surveys, including 18,741 women with a live birth; analytic samples were 15,552 women for maternal CoC and 7,625 children aged 12–23 months for full CoC. Wealth-related inequality was assessed using Erreygers concentration indices. Multivariable and multilevel logistic regression identified determinants of CoC completion, while Wagstaff, Oaxaca–Blinder, Fairlie, and inequality-of-opportunity decompositions identified inequality contributors.

**Results:**

Full CoC completion rose from 1.4% (2010) to 6.8% (2024); maternal CoC rose from 4.9% to 17.4%. Women completed an average of 8.3 of 11 interventions. Pro-rich inequality persisted for maternal CoC (Erreygers index = 0.036, *p* < 0.001); inequality in full CoC was smaller and not statistically significant (0.004, *p* = 0.497). Women's education, particularly higher education, was the strongest and most consistent predictor of completion (adjusted OR: 3.42 maternal, 3.45 full CoC, pooled). Contributors varied by method: education, wealth, and rural residence dominated the Wagstaff decomposition, while education, age, and parity contributed more consistently in the Oaxaca–Blinder and Fairlie decompositions. Circumstance variables reproduced the entirety of observed maternal CoC inequality, falling to 78.7% when wealth was excluded.

**Conclusions:**

Although MNCH CoC coverage improved substantially in Malawi, socioeconomic inequalities persisted, particularly for maternal CoC, and were closely linked to structural circumstances. Policies improving geographic access, expanding girls’ and women's education, and addressing structural barriers may support more equitable progress toward Sustainable Development Goal 3.

## Introduction

Despite substantial progress in reducing maternal and child mortality over recent decades, maternal, new-born, and child health (MNCH) remains a major public health challenge globally, particularly in sub-Saharan Africa (SSA). Between 2000 and 2020, the global maternal mortality ratio declined considerably; however, SSA still accounts for nearly 70% of maternal deaths worldwide (1). Similarly, although neonatal mortality has decreased over time, the pace of reduction remains insufficient to achieve Sustainable Development Goal (SDG) targets by 2030 (2). Malawi continues to face a significant burden of maternal and child mortality despite notable improvements in access to essential health services, highlighting the need for strategies that strengthen maternal and child survival across the entire reproductive and early childhood period (3, 4).

The continuum of care (CoC) framework has emerged as a cornerstone of maternal and child health programming because it promotes the delivery of integrated interventions from pregnancy through childbirth, the postnatal period, and early childhood (5). The framework emphasises that maternal and child health outcomes are maximised when women and children receive a complete package of services, including antenatal care, skilled birth attendance, postnatal care, immunisation, breastfeeding support, and family planning. Evidence from a recent systematic review and meta-analysis demonstrated that completion of the continuum of care significantly reduces maternal and neonatal mortality, while discontinuity at any stage increases the risk of adverse outcomes (5). Nevertheless, completion of the continuum remains low across many low- and middle-income countries. A pooled analysis of sub-Saharan African countries estimated that only 36% of women completed the maternal continuum of care (6), while a systematic review of African studies reported an average completion rate of only 20.9% (7).

Beyond low coverage, evidence increasingly shows that utilisation of continuum of care services is unequally distributed across socioeconomic groups. Multi-country studies conducted in SSA have consistently reported pro-rich inequalities in continuum of care completion, with women from wealthier households being substantially more likely to complete maternal health services than women from poorer households (8, 9). Similar patterns have been documented in Bangladesh, Ethiopia, and other low- and middle-income countries, where wealth, education, residence, and access to information strongly influence progression through the continuum of care (10–13). Studies further suggest that socioeconomic disadvantages accumulate across successive stages of care, leading to widening inequalities as women progress from antenatal care to delivery, postnatal care, and child health services (14, 15).

In Malawi, socioeconomic inequalities have been documented across several health outcomes and services, including HIV knowledge, cervical cancer screening, maternal healthcare utilisation, and childhood immunisation (16–21). Evidence also indicates persistent disparities in antenatal care utilisation and postnatal care uptake (22–26). However, most existing studies focus on individual maternal or child health interventions rather than continuity across the full maternal, newborn, and child health pathway. Furthermore, while regional studies have examined wealth-related inequalities in continuum of care completion, Malawi remains underrepresented in country-specific analyses investigating the magnitude and underlying drivers of socioeconomic inequality across the complete continuum of care (8, 9).

Understanding the extent and determinants of socioeconomic inequality in continuum of care completion is essential for designing equitable policies aimed at improving maternal and child health outcomes and achieving Universal Health Coverage. Although previous studies have identified factors associated with continuum of care utilisation, limited evidence exists on the drivers contributing to wealth-related inequalities in completion of the full maternal, newborn, and child health continuum of care in Malawi. This study addresses this gap by examining socioeconomic inequality in the completion of an eleven-component MNCH continuum of care using nationally representative data from the 2010, 2015–16, and 2024 Malawi Demographic and Health Surveys. Specifically, the study aimed to: (1) measure socioeconomic inequality and its severity in the completion of the continuum of care; (2) identify determinants of continuum of care completion; and (3) determine the factors contributing most to observed socioeconomic inequalities.

## Methods

### Study design and data source

This study utilised data from the 2010, 2015–16, and 2024 Malawi Demographic and Health Surveys (MDHS), nationally representative household surveys conducted by the National Statistical Office in collaboration with the Ministry of Health and ICF through the Demographic and Health Survey (DHS) Programme. The MDHS employs a stratified two-stage cluster sampling design. In the first stage, enumeration areas are selected from the national census sampling frame using probability proportional to size. In the second stage, households are systematically sampled within selected clusters. The surveys are designed to provide reliable estimates of health indicators at national, regional, and urban-rural levels. Detailed information regarding survey design and sampling procedures is available in the respective MDHS reports (27).

### Study population

The study population comprised women aged 15–49 years who had a live birth within the two years preceding each Malawi Demographic and Health Survey (MDHS). Consistent with previous continuum-of-care (CoC) studies (8, 9, 28), only the most recent live birth was retained to minimise recall bias and ensure comparability across survey rounds. After restricting to the most recent live birth of a child aged under 24 months and still alive at interview, 7,356 women were retained from the 2010 survey, 6,456 from the 2015–16 survey and 4,929 from the 2024 survey. Analyses of childhood vaccination and the complete 11-component continuum of care were further restricted to children aged 12–23 months (3,660 in 2010; 3,161 in 2015–16; 2,468 in 2024). Observations with missing values on any continuum component or study covariate were then excluded under a strict complete-case rule, yielding final analytical samples of 15,552 women for the maternal continuum of care (6,901 in 2010; 5,211 in 2015–16; 3,440 in 2024) and 7,625 children for the full continuum of care (3,442 in 2010; 2,523 in 2015–16; 1,660 in 2024). These complete-case samples were used throughout the analysis; the unweighted sample size for each estimate is reported in the corresponding tables, See Figure 1.

**Figure 1:**
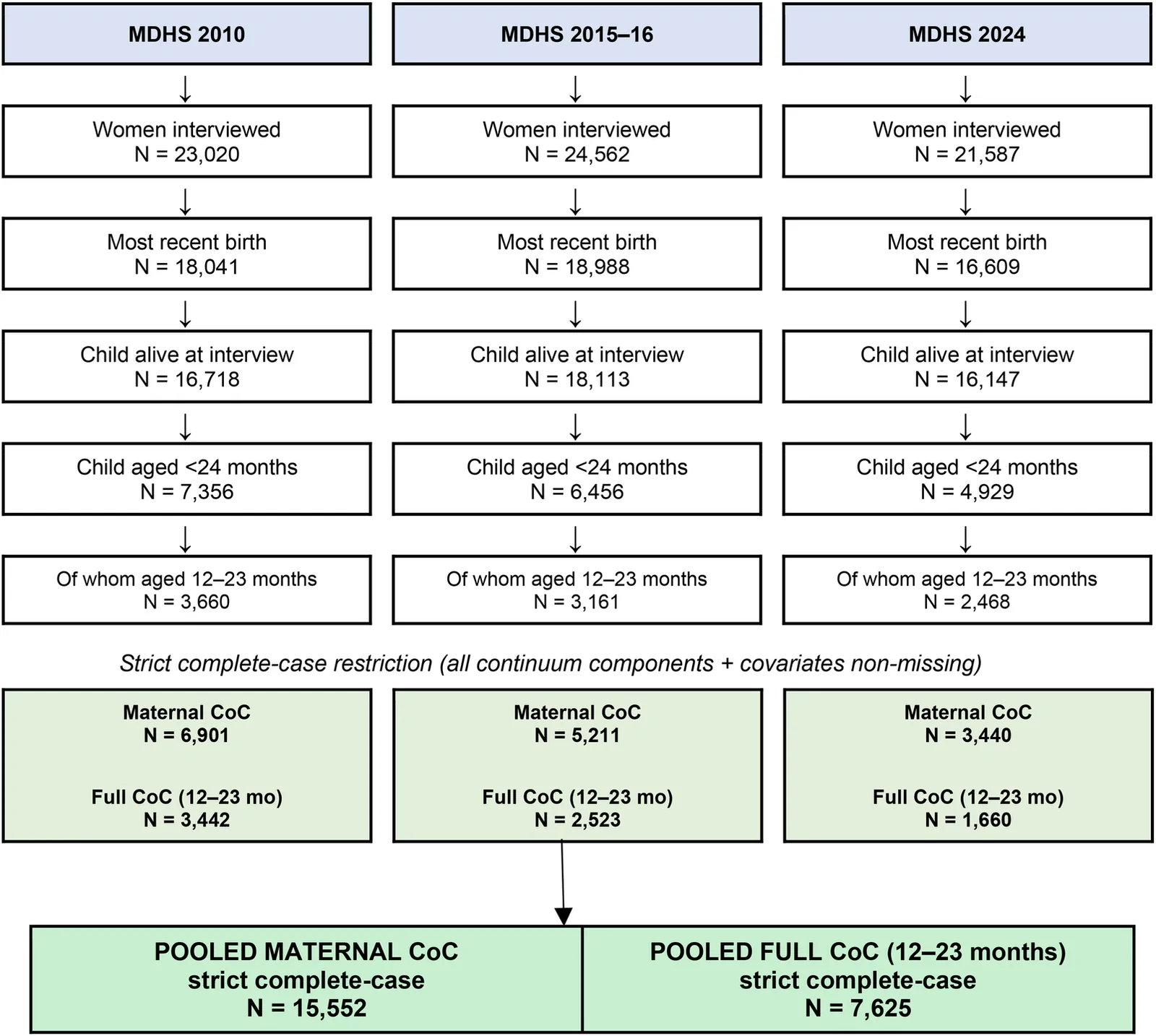
Participant flow diagram. Complete-case restriction required non-missing values on all continuum components (early ANC4+, tetanus, facility delivery, skilled attendance, maternal PNC ≤48 h, early breastfeeding, modern contraception, and — for full CoC — BCG, DPT3, Polio3, measles) and all analytical covariates (age, parity, woman's and husband's education, residence, region, wealth, distance to facility, radio/newspaper/TV exposure). These two pooled N's (15,552 and 7,625) are the denominators used in all tables and the Abstract.

### Outcome variable

The primary outcome was completion of the maternal, newborn, and child health (MNCH) continuum of care (CoC). Because there is no universally standardized measure of the MNCH continuum of care, the outcome was constructed using the composite coverage approach described in previous studies (28, 29). The continuum comprised eleven interventions: early ANC4+ (at least four antenatal visits with the first visit before the fourth month of pregnancy), ≥2 tetanus toxoid doses, facility delivery, skilled birth attendance, maternal postnatal care within 48 h, early initiation of breastfeeding, modern contraceptive use, BCG, DPT3, Polio3 and measles vaccination. Women and children aged 12–23 months who received all eleven interventions were coded 1 (complete); otherwise 0 (incomplete). The full continuum was assessed only among children aged 12–23 months. Newborn postnatal care was excluded because the DHS item was not comparable across survey rounds. A four-contact ANC threshold, commonly used in CoC studies (28), was retained for temporal comparability across the three surveys; sensitivity analyses using ≥8 contacts were conducted for the 2024 round (Supplementary Tables S1–S3).

### Explanatory variables

The explanatory variables were selected based on previous literature examining determinants of continuum of care utilisation and socioeconomic inequalities in maternal and child health service use (7, 8, 29, 30). Guided by the World Health Organization Social Determinants of Health Framework (31), Covariates were grouped into individual, household, and contextual factors. Individual-level variables included maternal age, parity, maternal educational attainment (no education, primary, secondary, and higher), and exposure to radio, television, and newspapers or magazines. Household-level variables included household wealth quintile, partner's educational attainment, and perceived distance to a health facility. Contextual factors comprised place of residence, region of residence, survey year, and the DHS primary sampling unit (PSU). Early antenatal care initiation was not included as a covariate because it is embedded in the early-ANC4+ component of the continuum-of-care outcomes.

### Measurement of socioeconomic inequality

Socioeconomic inequality in completion of the MNCH continuum of care was assessed using concentration curves and concentration indices (8, 9, 32). Concentration curves were constructed by plotting the cumulative proportion of continuum of care completion against the cumulative proportion of women ranked according to household wealth, beginning with the poorest and ending with the richest. A concentration curve below the line of equality indicates a pro-rich distribution, whereas a curve above the line indicates a pro-poor distribution. The standard concentration index (CI) was estimated as:

$$CI=\frac{2}{\mu }\mathrm{cov}(y_{i},R_{i})$$

where *μ* denotes the mean of the outcome variable, yᵢ represents continuum of care completion, and Rᵢ is the fractional rank of the individual in the wealth distribution. Because completion of the continuum of care is a binary outcome, the Erreygers corrected concentration index (ECI) was used as the primary measure of inequality to account for the bounded nature of the variable (32). The ECI was calculated as:

ECI=4μ×CI

Positive values indicate that completion of the continuum of care is concentrated among wealthier women, while negative values indicate concentration among poorer women. To examine the extent to which selected socioeconomic and geographic characteristics statistically reproduce the observed wealth-related inequality in maternal continuum of care (CoC), we estimated circumstances-only logistic regression models, following an inequality-of-opportunity approach (41). The full model included household wealth, place of residence, region, and partner's education. A second model excluded household wealth in order to assess how the reproduced inequality changed when wealth was omitted. Predicted probabilities from each model were obtained, and Erreygers concentration indices were calculated on these fitted probabilities using the survey design. The percentage of observed inequality that is statistically reproduced was defined as:

$$IOp=\left(\frac{CI_{circumstances}}{CI_{total}}\right)\times 100$$

where $CI_{circumstances}$ represents the concentration index of the predicted probabilities based on the selected circumstance variables and $CI_{total}$ represents the total observed concentration index. Higher values indicate a greater proportion of the observed inequality that is statistically reproduced by the selected circumstance variables.

### Determinants of continuum of care completion

To identify factors associated with completion of the continuum of care**,** logistic regression and multilevel logistic regression models were fitted. Survey-weighted logistic regression models were estimated separately for each survey round and for the pooled sample, with survey year included in the pooled model. The models accounted for the MDHS sampling weights, PSU clustering, and survey stratification. Because women and their children were nested within DHS primary sampling units (PSUs)/enumeration areas (clusters), a two-level random-intercept logistic regression model was estimated to account for clustering and between-PSU heterogeneity. The multilevel logistic model was specified as:

$$\ln\;\left[\frac{P_{ij}}{1-P_{ij}}\right]=\beta_{0}+\beta_{1}X_{1ij}+\beta_{2}X_{2ij}+\ldots +\beta_{k}X_{kij}+u_{j}$$

where Pᵢⱼ denotes the probability that woman i in cluster j completed the continuum of care, *β*₀ is the intercept, Xᵏᵢⱼ represents the explanatory variables, *β*ᵏ denotes the corresponding regression coefficients, and uⱼ represents the PSU-level random effect. Adjusted odds ratios (AORs) and 95% confidence intervals (CIs) were reported. Between-PSU variation was assessed using the intraclass correlation coefficient (ICC), calculated as:

$$ICC=\frac{\sigma_{u}^{2}}{\sigma_{u}^{2}+\frac{\pi^{2}}{3}}$$

where *σ*^2^ᵤ represents the PSU-level variance. The ICC quantifies the proportion of residual variation in completion of the continuum of care attributable to differences between PSUs.

### Decomposition of socioeconomic inequality

To identify the contributions of individual determinants to wealth-related inequality in continuum of care completion, the Erreygers concentration index (ECI) was decomposed using the Wagstaff decomposition approach (33). This method decomposes the observed inequality into the contributions of individual explanatory variables based on their association with continuum of care completion and their distribution across household wealth groups. Positive contributions indicate that a variable increases pro-rich inequality, whereas negative contributions indicate that it reduces inequality. To further examine wealth-related differences in continuum of care completion, Oaxaca–Blinder decomposition was used to decompose the observed rich–poor gap into components attributable to differences in population characteristics (endowments) and differences in the effects of those characteristics (coefficients). Because continuum of care completion is a binary outcome, Fairlie decomposition was also performed. The Fairlie approach extends the Oaxaca–Blinder decomposition to nonlinear models and estimates the contribution of individual explanatory variables to the observed gap between wealth groups (34).

### Statistical analysis

Descriptive statistics were used to summarise respondent characteristics and continuum of care completion. Concentration curves and Erreygers concentration indices were estimated to assess socioeconomic inequality. Survey-weighted logistic regression and multilevel logistic regression models were fitted to identify determinants of continuum of care completion. Wagstaff, Oaxaca–Blinder and Fairlie decompositions were conducted to identify drivers of socioeconomic inequality. All analyses accounted for the complex survey design. Within each survey round, sampling weights, primary sampling units and strata were applied as provided by DHS. After pooling, PSU and strata identifiers were made unique across rounds by grouping survey year with the original cluster and stratum codes; sampling weights were carried forward from each round without rescaling. Statistical significance was assessed at *p* < 0.05. Analyses were performed in Stata version 18.

### Ethical considerations

The study used publicly available secondary data obtained from the DHS Programme following formal approval. Ethical approval for the original surveys was granted by the Malawi National Health Sciences Research Committee and the Institutional Review Board of ICF. Written informed consent was obtained from all participants before data collection. The datasets were fully anonymised before being released for research purposes, and no personal identifiers were available to the investigators.

## Results

### Descriptive statistics and coverage trends

Table 1 summarises the socio-demographic and household characteristics of women included in the three Malawi Demographic and Health Surveys. The strict complete-case sample comprised 15,552 women (6,901 in 2010; 5,211 in 2015–16; and 3,440 in 2024). Across survey rounds, the educational profile of the sample changed: the proportion of women with no formal education declined from 16.2% in 2010 to 6.2% in 2024, while the proportion with secondary or higher education increased. The distribution of residence and region remained relatively stable; overall, 85.7% of women resided in rural areas and 43.9% lived in the Southern Region. In the pooled sample, 16.1% of women received early ANC4+, defined as at least four antenatal visits with the first visit occurring before the fourth month of pregnancy. Radio exposure was reported by 56.9% of women, newspaper exposure by 19.2% and television exposure by 15.9%. Perceived distance to a health facility was reported as a big problem by 56.9% of women. Mean age was 27.0 years (SE 0.07) and mean parity was 3.28 (SE 0.02).

**Table 1:** Socio-demographic and household characteristics of the study sample.

Characteristic	2010 *n* (%)	2015–16 *n* (%)	2024 *n* (%)	Pooled *n* (%)
*Household wealth quintile*				
Poorest	1,602 (22.1)	1,116 (23.2)	810 (26.0)	3,528 (23.3)
Poorer	1,570 (21.7)	1,207 (23.9)	589 (18.9)	3,366 (21.8)
Middle	1,555 (22.2)	1,040 (19.8)	602 (18.2)	3,197 (20.5)
Richer	1,279 (18.1)	929 (17.1)	643 (17.5)	2,851 (17.6)
Richest	895 (16.0)	919 (16.0)	796 (19.4)	2,610 (16.8)
*Woman's education*				
No education	1,083 (16.2)	593 (12.1)	182 (6.2)	1,858 (12.5)
Primary	4,850 (68.4)	3,494 (67.3)	2,258 (68.3)	10,602 (68.0)
Secondary	927 (14.5)	1,014 (18.7)	899 (23.1)	2,840 (17.8)
Higher	41 (0.9)	110 (2.0)	101 (2.5)	252 (1.6)
*Partner's education*				
No education	682 (10.7)	469 (10.1)	185 (6.4)	1,336 (9.5)
Primary	4,302 (61.0)	2,832 (54.9)	1,777 (54.6)	8,911 (57.5)
Secondary	1,792 (26.2)	1,664 (30.6)	1,284 (34.1)	4,740 (29.5)
Higher	125 (2.1)	246 (4.4)	194 (4.8)	565 (3.5)
*Place of residence*				
Urban	662 (14.6)	837 (13.7)	672 (14.7)	2,171 (14.3)
Rural	6,239 (85.4)	4,374 (86.3)	2,768 (85.3)	13,381 (85.7)
*Region*				
Northern	1,277 (11.7)	1,007 (12.3)	763 (14.5)	3,047 (12.6)
Central	2,421 (44.4)	1,837 (43.4)	1,124 (42.1)	5,382 (43.5)
Southern	3,203 (43.9)	2,367 (44.2)	1,553 (43.4)	7,123 (43.9)
*Radio exposure*				
No	1,866 (28.2)	2,666 (51.0)	2,075 (60.4)	6,607 (43.1)
Yes	5,035 (71.8)	2,545 (49.0)	1,365 (39.6)	8,945 (56.9)
*Newspaper exposure*				
No	5,346 (76.6)	4,362 (83.7)	2,942 (84.4)	12,650 (80.8)
Yes	1,555 (23.4)	849 (16.3)	498 (15.6)	2,902 (19.2)
*Television exposure*				
No	5,807 (84.0)	4,321 (84.3)	2,809 (84.2)	12,937 (84.1)
Yes	1,094 (16.0)	890 (15.7)	631 (15.8)	2,615 (15.9)
*Early ANC4+ (*≥*4 visits and first visit <4 months)*				
No	6,214 (90.4)	4,184 (81.4)	2,549 (74.9)	12,947 (83.9)
Yes	687 (9.6)	1,027 (18.6)	891 (25.1)	2,605 (16.1)
*Distance to health facility a big problem*				
Not a big problem	2,728 (42.1)	2,401 (43.3)	1,646 (45.0)	6,775 (43.1)
Big problem	4,173 (57.9)	2,810 (56.7)	1,794 (55.0)	8,777 (56.9)
*Continuous variables*				
Age in years, mean (SE)	27.2 (0.10)	26.9 (0.12)	26.8 (0.13)	27.0 (0.07)
Parity, mean (SE)	3.61 (0.04)	3.20 (0.04)	2.76 (0.04)	3.28 (0.02)
*Unweighted n*	6,901	5,211	3,440	15,552

Categorical variables are presented as unweighted *n* (survey-weighted %). Continuous variables are presented as survey-weighted mean (linearized standard error). All statistics are restricted to the strict complete-case maternal sample (*n* = 15,552).

Coverage of individual continuum-of-care components across survey rounds is presented in Table 2. ANC4+, defined as at least four visits with the first visit before the fourth month of pregnancy, increased from 9.6% to 25.1%. Facility-based delivery rose from 77.7% to 97.0%, skilled birth attendance from 75.3% to 96.3%, and maternal postnatal care within 48 h from 45.9% to 65.8%. Maternal continuum-of-care completion (early ANC4+, facility delivery and maternal postnatal care within 48 h) increased from 4.9% in 2010 to 17.4% in 2024. Full completion of all eleven continuum components among children aged 12–23 months increased from 1.4% to 6.8%. Early initiation of breastfeeding declined from 97.7% to 69.2%, and coverage of DPT3, Polio3 and measles vaccination also declined modestly, while BCG coverage remained high (>97%). Modern contraceptive use increased from 44.8% in 2010 to 73.8% in 2024.

**Table 2:** Coverage of individual continuum-of-care components, by survey round.

CoC component	2010 *n* (%)	2015–16 *n* (%)	2024 *n* (%)	Pooled *n* (%)
*Antenatal care*				
Early ANC4+ (≥4 visits and first visit <4 months)	687 (9.6)	1,027 (18.6)	891 (25.1)	2,605 (16.1)
≥2 tetanus toxoid doses	4,422 (66.6)	3,621 (70.3)	2,744 (79.7)	10,787 (70.8)
Facility-based delivery	5,318 (77.7)	4,910 (93.5)	3,339 (97.0)	13,567 (87.4)
Skilled birth attendance	5,162 (75.3)	4,825 (91.6)	3,315 (96.3)	13,302 (85.5)
Maternal postnatal check ≤48 h	3,065 (45.9)	2,403 (45.8)	2,371 (65.8)	7,839 (50.4)
Early initiation of breastfeeding	6,767 (97.7)	4,199 (77.8)	2,429 (69.2)	13,395 (84.6)
Modern contraceptive use	3,098 (44.8)	3,332 (64.6)	2,534 (73.8)	8,964 (58.0)
*Maternal CoC (early ANC4+ facility + PNC ≤48 h)*	326 (4.9)	526 (9.5)	651 (17.4)	1,503 (9.3)
*Childhood vaccination (children 12–23 months)*				
BCG	3,362 (97.6)	2,467 (97.9)	1,611 (97.5)	7,440 (97.7)
DPT3	3,252 (93.7)	2,375 (94.0)	1,445 (87.2)	7,072 (92.4)
Polio3	3,065 (86.8)	2,130 (83.2)	1,296 (78.3)	6,491 (83.7)
Measles	3,201 (93.2)	2,330 (91.7)	1,492 (89.8)	7,023 (91.9)
Full continuum of care	53 (1.4)	107 (3.8)	122 (6.8)	282 (3.4)
Unweighted *n* (maternal components)	6,901	5,211	3,440	15,552
Unweighted *n* (vaccination/full CoC)	3,442	2,523	1,660	7,625

Values are unweighted *n* (survey-weighted %). Maternal components use the strict complete-case maternal sample. Vaccination components and Full CoC use the strict complete-case full sample.

### Partial completion of the continuum of care

Table 3 shows the extent of partial completion of the eleven-component continuum of care among children aged 12–23 months. On average, children received 8.32 of the 11 recommended interventions, increasing from 7.99 components in 2010 to 8.78 components in 2024. The proportion completing at least eight components increased from 67.6% to 84.4%, and completion of at least ten components rose from 17.1% to 33.5% over the same period. Complete achievement of all eleven components increased from 1.4% in 2010 to 6.8% in 2024.

**Table 3:** Partial completion of the continuum of care among children aged 12–23 months.

Measure	2010	2015–16	2024	Pooled
Completed ≥8 of 11 components, *n* (%)	2,281 (67.6)	2,044 (79.7)	1,419 (84.4)	5,744 (75.3)
Completed ≥10 of 11 components, *n* (%)	604 (17.1)	606 (22.4)	583 (33.5)	1,793 (22.5)
Completed all 11 components, *n* (%)	53 (1.4)	107 (3.8)	122 (6.8)	282 (3.4)
Mean number of components completed (SE)	7.99 (0.04)	8.46 (0.04)	8.78 (0.04)	8.32 (0.02)
*Unweighted n*	3,442	2,523	1,660	7,625

Values are unweighted *n* (survey-weighted %) except mean (SE). Strict complete-case full CoC analytic sample (*N* = 7,625).

### Socioeconomic inequality in CoC completion

Table 4 presents the Erreygers concentration indices for individual continuum-of-care components, maternal continuum of care, and full continuum of care. Maternal continuum of care exhibited significant pro-rich inequality, with an ECI of 0.0357 (95% CI: 0.0209–0.0504; *p* < 0.001). Among individual maternal services, maternal postnatal care within 48 h showed the largest pro-rich inequality (ECI = 0.1247), followed by skilled birth attendance (ECI = 0.0954), facility-based delivery (ECI = 0.0921), and early ANC4+ (ECI = 0.0310), all statistically significant at *p* < 0.001. Modern contraceptive use also showed significant pro-rich inequality (ECI = 0.0511; *p* < 0.001). In contrast, inequality in tetanus toxoid vaccination was not statistically significant (ECI = 0.0186; *p* = 0.069), while early initiation of breastfeeding showed a small, statistically non-significant pro-poor concentration (ECI = −0.0163; *p* = 0.094). Among childhood vaccination indicators, BCG (ECI = 0.0112; *p* = 0.019) and measles vaccination (ECI = 0.0252; *p* = 0.003) showed small but statistically significant pro-rich inequalities, whereas DPT3 and Polio3 were not statistically significant. The full continuum of care showed a small and non-significant pro-rich inequality (ECI = 0.0038; *p* = 0.497). The corresponding concentration curves for early ANC4+, facility-based delivery and maternal postnatal care within 48 h are shown in Figures 2–4; each lies below the line of equality, consistent with the pro-rich concentration indices in Table 4.

**Figure 2:**
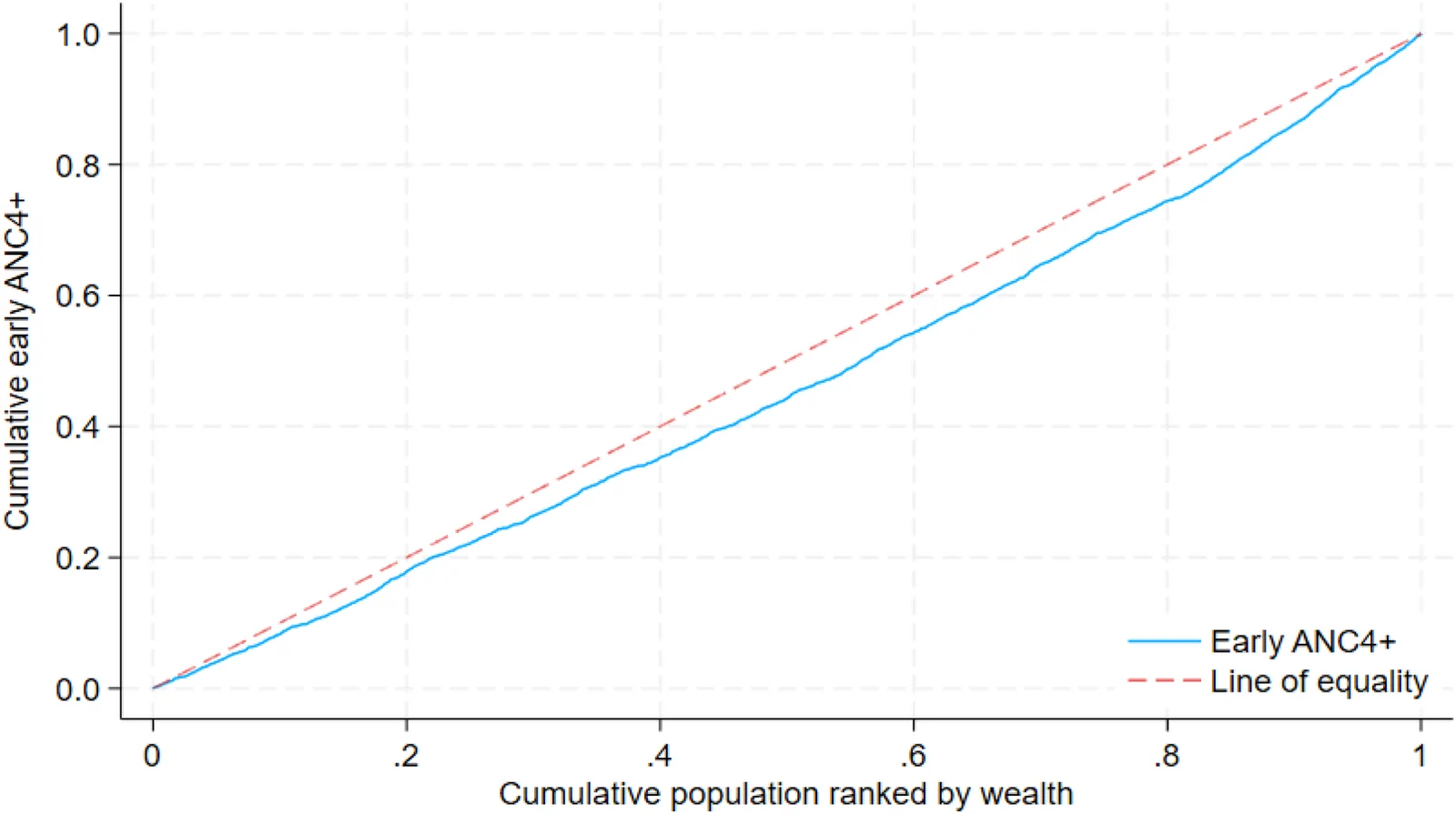
Concentration curve for early ANC4+ (≥4 visits with first visit before the fourth month), pooled.

**Figure 3:**
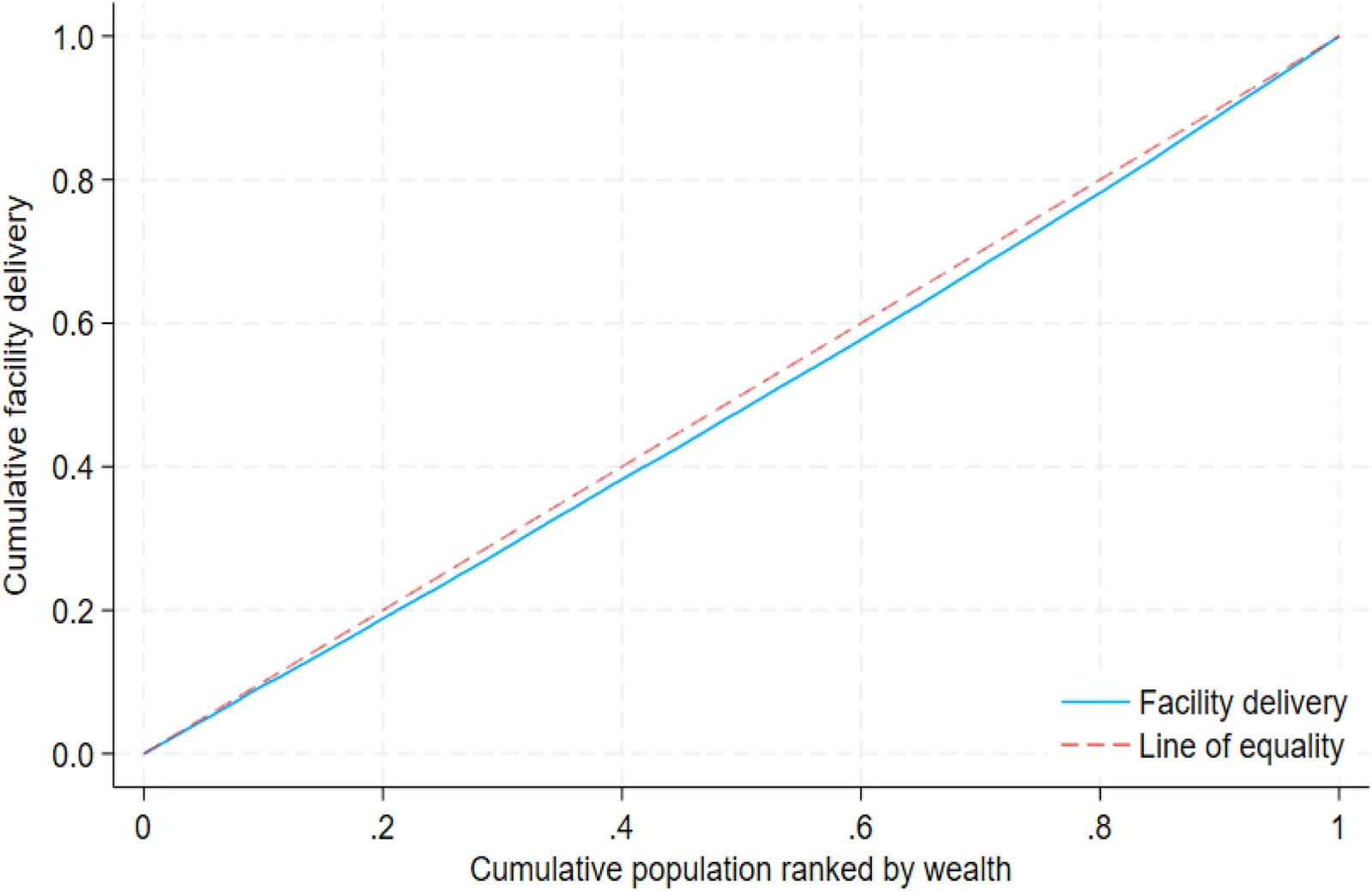
Concentration curve for facility delivery.

**Figure 4:**
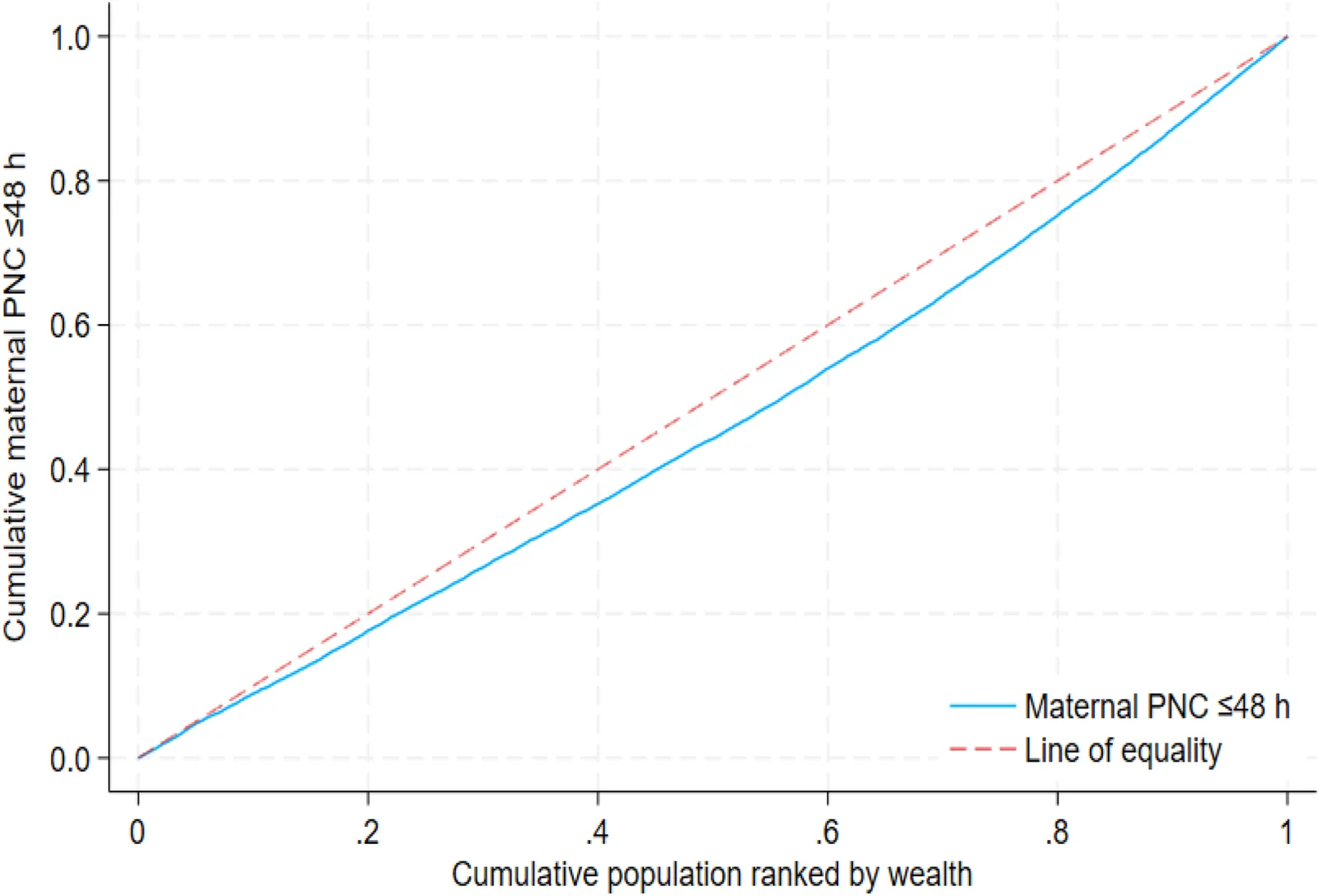
Concentration curve for maternal postnatal care within 48 hours.

**Table 4:** Erreygers concentration indices for continuum-of-care components.

Indicator	N	Erreygers CI (95% CI)	*p*-value
Early ANC4+ (≥4 ANC visits, first visit <4 months)	15,552	0.0310 (0.0132–0.0488)	<0.001
≥2 tetanus toxoid doses	15,552	0.0186 (−0.0014–0.0386)	0.069
Facility-based delivery	15,552	0.0921 (0.0764–0.1077)	<0.001
Skilled birth attendance	15,552	0.0954 (0.0784–0.1123)	<0.001
Maternal postnatal care ≤48 h	15,552	0.1247 (0.1001–0.1493)	<0.001
Early initiation of breastfeeding	15,552	−0.0163 (−0.0354–0.0027)	0.094
Modern contraceptive use	15,552	0.0511 (0.0272–0.0751)	<0.001
Maternal continuum of care	15,552	0.0357 (0.0209–0.0504)	<0.001
*Childhood vaccination (12–23 months)*			
BCG	7,625	0.0112 (0.0019–0.0206)	0.019
DPT3	7,625	−0.0005 (−0.0182–0.0171)	0.954
Polio3	7,625	0.0140 (−0.0112–0.0391)	0.277
Measles	7,625	0.0252 (0.0084–0.0419)	0.003
Full continuum of care (11 components)	7,625	0.0038 (−0.0072–0.0149)	0.497

Erreygers normalised concentration index. Positive values indicate pro-rich inequality. 95% confidence intervals calculated as Index ± 1.96 × standard error. All estimates use the strict complete-case samples.

Table 5 presents wealth-related inequality in maternal continuum of care across survey rounds. Maternal continuum of care consistently exhibited significant pro-rich inequality throughout the study period, with the Erreygers concentration index ranging from 0.0266 in 2010 to 0.0606 in 2024 (all *p* ≤ 0.028). Inequality remained broadly stable between 2010 and 2015–16, then increased in 2024. The pooled estimate was 0.0357 (95% CI: 0.0209–0.0504; *p* < 0.001), indicating persistent socioeconomic inequality in maternal continuum-of-care completion. Figure 5 displays these temporal trends.

**Table 5:** Erreygers concentration indices for maternal CoC, by survey year.

Survey year	N	Erreygers CI (95% CI)	*p*-value
*Maternal CoC*			
2010	6,901	0.0266 (0.0087–0.0444)	0.004
2015–16	5,211	0.0296 (0.0032–0.0561)	0.028
2024	3,440	0.0606 (0.0257–0.0956)	<0.001
Pooled	15,552	0.0357 (0.0209–0.0504)	<0.001
*Full CoC*			
2010	3,442	0.0091 (−0.0012–0.0193)	0.085
2015–16	2,523	−0.0079 (−0.0297–0.0140)	0.482
2024	1,660	0.0105 (−0.0189–0.0398)	0.485
Pooled	7,625	0.0038 (−0.0072–0.0149)	0.497

Strict complete-case maternal sample. Positive values indicate pro-rich inequality.

**Figure 5:**
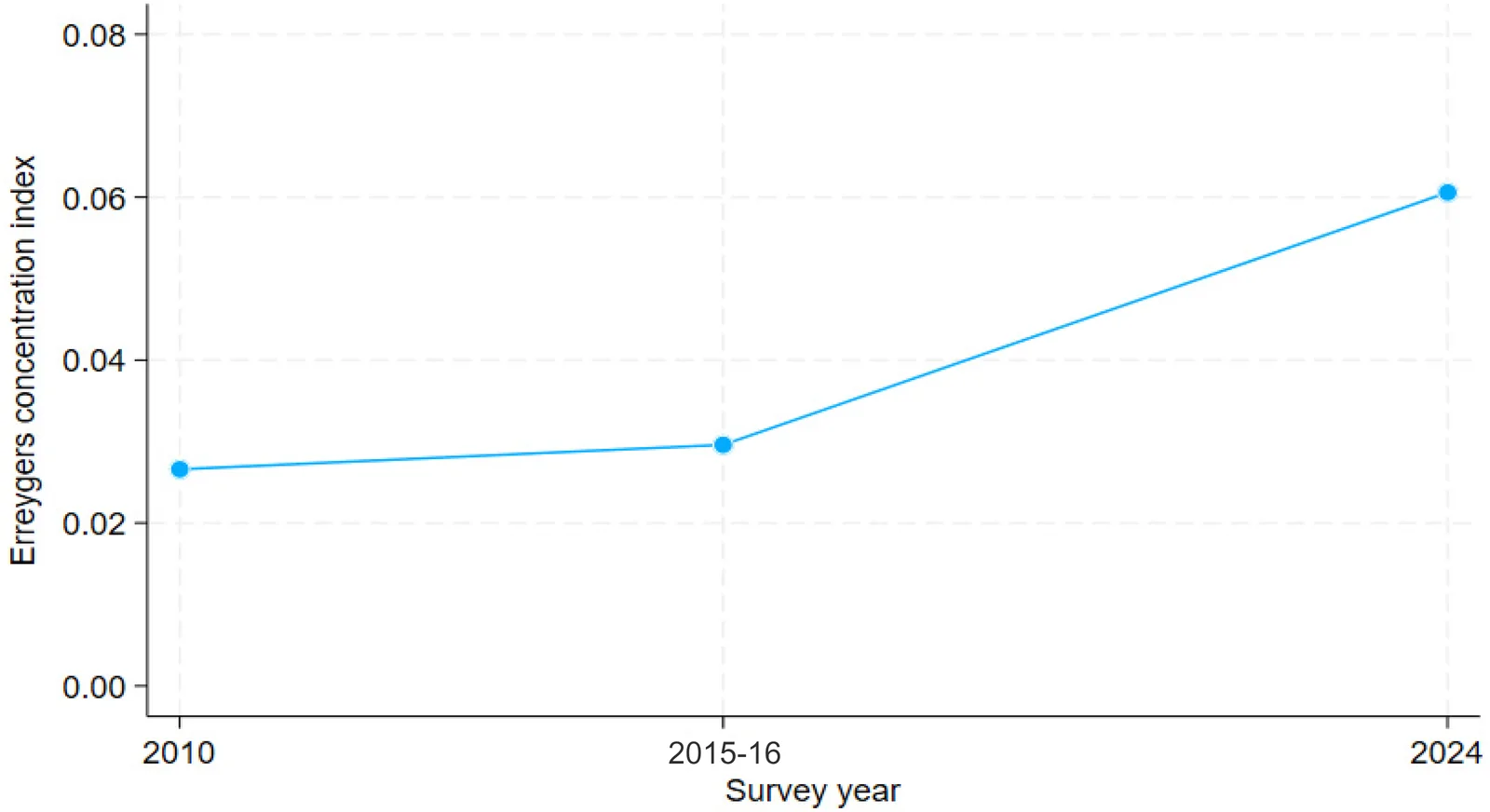
Trends in the Erreygers concentration index for maternal continuum of care, Malawi DHS 2010–2024.

Table 6 presents the Erreygers concentration indices for predicted maternal CoC probabilities from the circumstances-only models. The index from the full model that included household wealth, residence, region, and partner's education was 0.0357 (95% CI 0.0338–0.0375), reproducing 100.0% of the observed wealth-related inequality. When household wealth was excluded, the predicted index fell to 0.0281 (95% CI 0.0261–0.0300), corresponding to 78.7% of the observed inequality. These findings indicate that the selected variables jointly reproduce a large share of the observed socioeconomic gradient in maternal CoC, with wealth making a substantial contribution within this model specification.

**Table 6:** Erreygers concentration indices for predicted maternal CoC probabilities under circumstances-only models.

Model	N	Erreygers CI (95% CI)	*p*-value	% of observed inequality reproduced
Full circumstances model (wealth, residence, region, partner's education)	15,552	0.0357 (0.0338–0.0375)	<0.001	100.0%
Circumstances model excluding wealth (residence, region, partner's education)	15,552	0.0281 (0.0261–0.0300)	<0.001	78.7%

Predicted probabilities are fitted values from survey-weighted logistic regressions of maternal CoC on the listed variables. Erreygers concentration indices were estimated on the fitted probabilities under the survey design (bounded, limits 0–1). 95% confidence intervals were calculated as Index ± 1.96 × standard error. Percentage of observed inequality reproduced = predicted Erreygers CI ÷ observed pooled maternal CoC Erreygers CI (0.0357) × 100. Strict complete-case maternal sample. The analysis shows the share of the observed wealth-related inequality that is statistically reproduced by the included variables.

### Determinants of CoC completion

Table 7 presents the survey-weighted multivariable logistic regression results for completion of maternal continuum of care (CoC) across the three DHS rounds and the pooled sample. Women's education, particularly attainment of higher education, was the strongest and most consistent predictor of completion of maternal CoC, with markedly greater odds of completion in every survey round and in the pooled model (AOR=3.70, 2010; 3.85, 2015–16; 2.77, 2024; 3.42, pooled; all *p* < 0.05 or below). Secondary education was associated with higher odds of completion in the pooled model (AOR=1.50, *p* < 0.05) but not significantly in any single round. Partner's education showed a more sporadic pattern, with partner's primary education positively associated with completion in 2010 only (AOR=2.24), and partner's higher education only borderline significant in 2010 and in the pooled model. The relationship between household wealth and completion was inconsistent across rounds: in 2010, women in the richer quintile had significantly higher odds of completion than the poorest (AOR=1.70), whereas in 2015–16 the middle and richest quintiles were instead associated with significantly *lower* odds (AOR=0.68 and 0.62, respectively); no wealth gradient was evident in 2024 or in the pooled model. Perceiving distance to a health facility as a big problem was associated with lower odds of completion in 2024 (AOR=0.79) and in the pooled sample (AOR=0.83), though not in the earlier rounds. Radio, newspaper, and television exposure showed no significant association with completion in any round or in the pooled model. Regional disparities were evident from 2015 to 16 onward, with women in the Central and Southern regions having significantly lower odds of completion than those in the Northern Region in 2015–16, 2024, and the pooled model, though this did not hold in 2010. Parity was negatively associated with completion in 2010 and in the pooled model, while age was positively associated only in the pooled model. In the pooled analysis, women surveyed in 2015–16 and 2024 had significantly higher odds of completing maternal CoC than those surveyed in 2010 (AOR=1.93 and 3.64, respectively), reflecting improvement over time after adjustment for other covariates.

**Table 7:** Adjusted odds ratios for maternal continuum of care.

Variable/category	2010	2015–16	2024	Pooled
Wealth quintile				
*Poorest (ref.)*				
Poorer	1.07 (0.65–1.76)	0.75 (0.52–1.09)	1.13 (0.78–1.64)	0.96 (0.76–1.22)
Middle	1.29 (0.79–2.11)	0.68 (0.46–1.00)*	0.99 (0.70–1.40)	0.94 (0.74–1.18)
Richer	1.70 (1.08–2.69)*	0.81 (0.57–1.16)	1.23 (0.83–1.81)	1.16 (0.92–1.46)
Richest	1.30 (0.68–2.47)	0.62 (0.39–0.99)*	1.15 (0.76–1.75)	0.97 (0.72–1.29)
Woman's education				
*No education (ref.)*				
Primary	1.10 (0.68–1.79)	1.44 (0.96–2.15)	1.15 (0.69–1.92)	1.23 (0.94–1.60)
Secondary	1.52 (0.80–2.90)	1.60 (0.95–2.69)	1.46 (0.81–2.63)	1.50 (1.08–2.08)*
Higher	3.70 (1.39–9.88)**	3.85 (1.50–9.87)**	2.77 (1.17–6.58)*	3.42 (1.98–5.90)***
Partner's education				
*No education (ref.)*				
Primary	2.24 (1.21–4.13)*	1.08 (0.64–1.81)	0.94 (0.58–1.54)	1.22 (0.90–1.66)
Secondary	1.76 (0.86–3.61)	1.19 (0.68–2.08)	0.86 (0.52–1.42)	1.14 (0.82–1.59)
Higher	2.93 (0.92–9.35)	1.75 (0.82–3.74)	1.04 (0.52–2.06)	1.59 (0.98–2.59)
Residence				
*Urban (ref.)*				
Rural	0.96 (0.48–1.92)	0.94 (0.66–1.34)	1.18 (0.85–1.64)	1.01 (0.77–1.34)
Distance to facility				
*Not a problem (ref.)*				
Big problem	0.92 (0.66–1.30)	0.82 (0.64–1.05)	0.79 (0.64–0.98)*	0.83 (0.72–0.96)*
Radio exposure				
*No (ref.)*				
Yes	1.03 (0.71–1.49)	1.24 (0.93–1.64)	1.07 (0.84–1.37)	1.14 (0.96–1.34)
Newspaper exposure				
*No (ref.)*				
Yes	0.70 (0.44–1.09)	0.87 (0.62–1.23)	1.14 (0.86–1.52)	0.92 (0.75–1.12)
TV exposure				
*No (ref.)*				
Yes	0.92 (0.62–1.37)	1.36 (0.97–1.92)	1.22 (0.92–1.64)	1.14 (0.94–1.40)
Age/parity				
Age (years)	1.03 (0.98–1.08)	1.03 (0.99–1.07)	1.01 (0.98–1.04)	1.02 (1.00–1.05)*
Parity	0.82 (0.70–0.96)*	0.91 (0.80–1.03)	1.01 (0.91–1.11)	0.91 (0.84–0.97)**
Region				
*Northern (ref.)*				
Central	0.98 (0.63–1.53)	0.71 (0.52–0.98)*	0.70 (0.52–0.94)*	0.75 (0.62–0.92)**
Southern	1.11 (0.76–1.63)	0.63 (0.47–0.85)**	0.71 (0.53–0.96)*	0.76 (0.63–0.92)**
Survey year				
*2010 (ref.)*				
2015–16				1.93 (1.54–2.42)***
2024				3.64 (2.88–4.58)***
N	**6,901**	**5,211**	**3,440**	**15,552**

Values are adjusted odds ratios (AORs) with 95% confidence intervals in brackets. Survey-weighted logistic regression (svy: logit). Outcome: completion of the maternal continuum of care. The pooled model additionally adjusts for survey round. Reference categories are shown in parentheses. * *p* < 0.05; ** *p* < 0.01; *** *p* < 0.001.

The multilevel mixed-effects logistic regression results for completion of maternal CoC are presented in Table 8. The results show that higher education remained the strongest individual-level predictor, with women who had attained higher education having more than twice the odds of completion compared with those with no education (AOR=2.13, 95% CI: 1.35–3.34). Partner's higher education was similarly associated with greater odds of completion (AOR=1.81, 95% CI: 1.22–2.66), while partner's primary and secondary education were only marginally associated. Household wealth was not significantly associated with completion in the multilevel model, nor was rural residence. Perceiving distance to a facility as a big problem and radio exposure were both marginally associated with completion (AOR=0.88 and 1.14, respectively, *p* < 0.10). Age was positively associated with completion (AOR=1.03, *p* < 0.001) while parity was negatively associated (AOR=0.90, *p* < 0.001). Women in the Central and Southern regions had significantly lower odds of completion than those in the Northern Region (AOR=0.83 and 0.81, respectively). The odds of completion increased substantially over time, with women surveyed in 2015–16 and 2024 having more than twice and over four times the odds of completion, respectively, relative to 2010 (AOR=2.18 and 4.41). The intraclass correlation coefficient indicated that 12.2% of the residual variation in completion of maternal CoC was attributable to differences between DHS primary sampling units, pointing to a meaningful degree of cluster-level heterogeneity not captured by the individual- and household-level covariates.

**Table 8:** Multilevel logistic regression.

Variable/category	AOR (95% CI)
*Wealth quintile*	
Poorest (ref.)	
Poorer	0.94 (0.78–1.14)
Middle	1.03 (0.85–1.24)
Richer	1.12 (0.92–1.37)
Richest	1.14 (0.90–1.45)
*Woman's education*	
No education (ref.)	
Primary	1.10 (0.87–1.39)
Secondary	1.22 (0.93–1.61)
Higher	2.13 (1.35–3.34)**
*Partner's education*	
No education (ref.)	
Primary	1.24 (0.96–1.61)
Secondary	1.26 (0.96–1.67)
Higher	1.81 (1.22–2.66)**
*Residence*	
Urban (ref.)	
Rural	0.93 (0.76–1.13)
*Distance to facility*	
Not a problem (ref.)	
Big problem	0.88 (0.78–1.00)*
*Media exposure*	
Radio (yes vs. no)	1.14 (1.00–1.30)
Newspaper (yes vs. no)	1.09 (0.93–1.27)
TV (yes vs. no)	1.11 (0.94–1.31)
*Age/parity*	
Age (years)	1.03 (1.01–1.05)***
Parity	0.90 (0.85–0.95)***
*Region*	
Northern (ref.)	
Central	0.83 (0.70–0.99)*
Southern	0.81 (0.68–0.96)*
*Survey year*	
2010 (ref.)	
2015–16	2.18 (1.84–2.58)***
2024	4.41 (3.69–5.27)***
*Random effects*	
Random-intercept variance (PSU)	0.456 (0.330–0.629)
ICC	0.122 (0.091–0.160)
*N (clusters)*	**15,552 (2,455)**

Values are adjusted odds ratios (AORs) with 95% confidence intervals. Reference categories are shown in parentheses. *p *<* 0.05; **p *<* 0.01; ***p *<* *0.001. Models include random intercepts at the DHS primary sampling unit (PSU)/enumeration area (EA) level.*

### Drivers of socioeconomic inequality

The Wagstaff decomposition results are presented in Table 9, separately for maternal CoC and full CoC completion. For full CoC, women's secondary education made by far the largest positive contribution to pro-rich inequality (162.7% of the concentration index), followed by partner's secondary education (80.6%) and partner's higher education (76.4%); the poorer wealth quintile also contributed positively (62.1%). These positive contributions were substantially offset by rural residence, which made the largest negative contribution (−106.6%), along with women's primary education (−67.0%) and television exposure (−59.5%), both of which reduced the observed pro-rich inequality. For maternal CoC, women's higher and secondary education were again the largest positive contributors (27.0% and 24.4%, respectively), followed by partner's higher education (14.6%) and the richer wealth quintile (11.4%); television exposure (10.1%), radio exposure (8.8%), and perceived distance to a facility (8.2%) each made smaller positive contributions. Unlike the earlier decomposition, the residual (unexplained) component was negligible for both outcomes, indicating that the observed pro-rich inequality in CoC completion is now almost fully accounted for by the variables included in the model. Because several individual contributions exceed 100% of the total concentration index in absolute value, particularly for full CoC, these should be read as the relative direction and weight of each factor rather than as literal shares of a bounded total, since positive and negative contributions offset one another.

**Table 9:** Wagstaff decomposition of the concentration index for maternal and full CoC completion.

Variable	Maternal CoC		Full CoC	
	Contribution	% of CI	Contribution	% of CI
Age	+0.00417	+4.3	−0.00124	−4.4
Parity	+0.00577	+6.0	−0.00190	−6.7
Wealth: poorer	+0.00178	+1.9	+0.01755	+62.1
Wealth: middle	−0.00120	−1.3	−0.00431	−15.3
Wealth: richer	+0.01093	+11.4	−0.01043	−36.9
Wealth: richest	−0.00433	−4.5	−0.04593	−162.7
Woman's educ.: primary	−0.00576	−6.0	−0.01891	−67.0
Woman's educ.: secondary	+0.02346	+24.4	+0.04593	+162.7
Woman's educ.: higher	+0.02588	+27.0	+0.01184	+41.9
Partner's educ.: primary	−0.00922	−9.6	+0.01409	+49.9
Partner's educ.: secondary	+0.00446	+4.7	+0.02275	+80.6
Partner's educ.: higher	+0.01401	+14.6	+0.02158	+76.4
Rural residence	−0.00066	−0.7	−0.03010	−106.6
Distance a big problem	+0.00788	+8.2	+0.00794	+28.1
Radio exposure	+0.00848	+8.8	+0.00933	+33.1
Newspaper exposure	−0.00434	−4.5	−0.00183	−6.5
TV exposure	+0.00968	+10.1	−0.01680	−59.5
Region: Central	+0.00698	+7.3	+0.00918	+32.5
Region: Southern	−0.00184	−1.9	−0.00021	−0.7
Survey round: 2015–16	−0.00223	−2.3	−0.00376	−13.3
Survey round: 2024	+0.00205	+2.1	+0.00346	+12.3
*Residual (unexplained)*	**−0**.**00000**	**−0**.**0**	**0**.**00000**	**0**.**0**
*Total concentration index*	**0**.**09595**	**100**.**0**	**0**.**02823**	**100**.**0**

Contributions were estimated using the Wagstaff decomposition of the concentration index. Percentage contribution was calculated as % Contribution to CI = (Contribution/CI) × 100, where the total concentration index (CI) is 0.09595 for maternal CoC and 0.02823 for full CoC, as shown in the table. Positive values indicate determinants that increase pro-rich inequality, whereas negative values indicate determinants that reduce it. Individual percentage contributions may exceed 100% in absolute value because positive and negative contributions offset one another. The residual (unexplained) component is calculated as the difference between the total concentration index and the sum of the explained contributions.

The Oaxaca–Blinder decomposition presented in Table 10 separates the observed wealth gap into differences in characteristics (endowments) and differences in the effects of those characteristics (coefficients). For maternal CoC, the not-rich group had a substantially lower mean completion rate than the rich group (8.0% versus 11.8%), a gap of 3.8 percentage points that was highly significant. Differences in endowments accounted for about two-thirds of this gap (−0.0260 of −0.0377, *p* < 0.001), with the coefficients component also significant (−0.0334, *p* < 0.05) and partly offset by a marginally significant interaction term. Among the detailed endowments, age, parity, and television exposure were significant contributors, while women's secondary or higher education was only marginally so; within the coefficients component, age, parity, and television exposure again stood out. For full CoC, the overall gap between the not-rich and rich groups (3.2% versus 3.8%) was small and not statistically significant, and none of the detailed endowment contributions reached significance. Notably, the coefficients (unexplained) component was itself significant (−0.0124, *p* < 0.05), suggesting that differences in the returns to characteristics between wealth groups persist even though the overall completion gap could not be statistically distinguished from zero.

**Table 10:** Oaxaca–Blinder decomposition of the gap in CoC coverage.

Component/variable	Maternal CoC	Full CoC
*Overall*		
Mean — not-rich (G0)	0.0800 (0.0033)***	0.0317 (0.0029)***
Mean — rich (G1)	0.1177 (0.0055)***	0.0381 (0.0043)***
Difference (G0−G1)	−0.0377 (0.0064)***	−0.0064 (0.0052)
Endowments (explained)	−0.0260 (0.0054)***	−0.0041 (0.0043)
Coefficients (unexplained)	−0.0334 (0.0134)*	−0.0124 (0.0058)*
Interaction	0.0217 (0.0125)	0.0101 (0.0054)
*Endowments (detailed)*		
Woman's educ.: secondary+	−0.0070 (0.0040)	−0.0039 (0.0035)
Rural residence	0.0033 (0.0042)	0.0053 (0.0051)
Age	−0.0061 (0.0016)***	−0.0015 (0.0013)
Parity	−0.0106 (0.0023)***	−0.0014 (0.0010)
Radio exposure	0.0027 (0.0028)	−0.0024 (0.0024)
Newspaper exposure	0.0031 (0.0021)	0.0013 (0.0017)
TV exposure	−0.0089 (0.0027)**	−0.0002 (0.0020)
Distance a big problem	−0.0025 (0.0021)	−0.0011 (0.0019)
*Coefficients (detailed)*		
Woman's educ.: secondary+	0.0059 (0.0080)	0.0027 (0.0028)
Rural residence	0.0149 (0.0308)	0.0105 (0.0061)
Age	−0.1534 (0.0672)*	−0.0099 (0.0223)
Parity	0.0771 (0.0308)*	0.0041 (0.0087)
Radio exposure	−0.0037 (0.0120)	−0.0108 (0.0059)
Newspaper exposure	0.0028 (0.0061)	0.0012 (0.0023)
TV exposure	−0.0153 (0.0074)*	−0.0047 (0.0029)
Distance a big problem	−0.0049 (0.0074)	−0.0001 (0.0030)
Constant	0.0431 (0.0700)	−0.0054 (0.0207)
*N (not-rich/rich)*	**10,091/5,461**	**4,968/2,657**

Endowments = explained by differences in characteristics; Coefficients = unexplained (returns to characteristics). Interaction shown in overall panel only. Secondary + = secondary or higher education; Distance = big problem getting medical help due to distance; Early ANC = early antenatal care.

*p < 0.05.

**p < 0.01.

***p < 0.001.

The Fairlie non-linear decomposition results in Table 11 further examine how differences in observed characteristics contributed to the wealth gap in CoC completion. For maternal CoC, the total explained component accounted for only a modest share of the overall gap (−0.0043, or 11.3% of the −0.0377 difference), with women's secondary or higher education the only significant individual contributor (−0.0102, *p* < 0.05); age, radio exposure, and perceived distance to a facility were marginally significant. For full CoC, the total explained component (+0.0060) ran in the opposite direction to the overall gap (−0.0064), implying that differences in observed characteristics alone would have predicted a *smaller* completion rate among the rich than the not-rich, rather than the modestly higher rate actually observed; rural residence and radio exposure were the significant individual contributors to this explained component (0.0071 and 0.0048, respectively), with women's secondary or higher education and television exposure only marginally significant.

**Table 11:** Fairlie non-linear decomposition of the gap in CoC coverage.

Component/variable	Maternal CoC	Full CoC
Pr(complete | not-rich)	0.0800	0.0317
Pr(complete | rich)	0.1177	0.0381
*Difference*	−0.0377	−0.0064
*Total explained*	−0.0043 (11.3% of gap)	+0.0060 (opposite sign)
*Contribution of characteristics*		
Woman's educ.: secondary+	−0.0102 (0.0044)*	−0.0084 (0.0044)
Rural residence	0.0073 (0.0097)	0.0071 (0.0023)**
Age	−0.0043 (0.0024)	−0.0012 (0.0020)
Parity	−0.0005 (0.0011)	0.0003 (0.0010)
Radio exposure	0.0034 (0.0019)	0.0048 (0.0021)*
Newspaper exposure	0.0014 (0.0017)	0.0000 (0.0012)
TV exposure	0.0010 (0.0027)	0.0037 (0.0019)
Distance a big problem	−0.0022 (0.0012)	−0.0004 (0.0004)
*N (not-rich/rich)*	**10,091/5,461**	**4,968/2,657**

Positive contribution widens the observed gap; negative contribution narrows it. Total explained is the sum of individual contributions. Unexplained portion = overall difference−total explained.

*p < 0.05

***p < 0.001.

## Discussion

This study utilized three rounds of nationally representative DHS data (2010–2024) to examine the magnitude of socioeconomic inequality in MNCH continuum of care completion, identify the determinants of continuum of care completion, and determine the factors contributing most to observed socioeconomic inequalities. Over this period, completion of the full eleven-component continuum nearly doubled, and maternal continuum completion also rose substantially. This progress coincides with the expansion of national maternal and child health programs and community-based service delivery (27). Despite these gains, pro-rich inequality in maternal CoC completion persisted throughout the study. In contrast, inequality in the full continuum was not statistically significant in any survey round or in the pooled analysis, suggesting that broader access to the continuum was comparatively more equitable throughout the study period. However, completion of services requiring repeated contact with the health system remained socioeconomically patterned. This finding provides only partial support for the inverse equity hypothesis, which posits that newly expanded services are first adopted by wealthier groups before spreading more evenly (11).

Given that overall completion of the full continuum remained modest, it is important to identify points of care discontinuation. Most women initiated contact with the health system through skilled antenatal care (ANC) and facility delivery. However, completion rates declined for repeated ANC visits, timely postnatal care, and childhood immunization. This pattern, where women initiate but do not complete the recommended package of care, has been observed by (9). This contrasts with countries like Lao PDR (35), The Gambia (36), and Ghana (37), where overall completion remained very low from the outset. In Malawi, discontinuity is concentrated in the later stages of the continuum, which may reflect the cumulative burden of repeated facility contact, competing household responsibilities, and the travel and opportunity costs associated with multiple visits (38).

These determinants align with Andersen's Behavioral Model of health service use. Women's education, particularly higher education, emerged as the strongest and most consistent predictor of both maternal and full CoC completion, underscoring the pivotal role of educational attainment in sustained engagement across the continuum. More educated women may have greater opportunities to access health information, navigate the health system, and follow through on recommended contacts, health education, and referrals to other services (14, 23, 39). Perceived distance to a facility and partner's education were also independently associated with completion. The DHS measure of perceived distance captures a woman's belief that reaching a facility is problematic, rather than actual travel distance. This likely reflects a combination of physical accessibility, transport availability, cost, and prior experience with the referral system, rather than distance alone. This is consistent with evidence on referral delays and transport constraints within Malawi's rural health system (38, 40). These findings align with the WHO Social Determinants of Health framework, which identifies education, wealth, and geographic access as fundamental influences on health opportunity (31). The inequality-of-opportunity analysis further reinforced this structural interpretation: circumstance variables (household wealth, residence, region, and partner's education) statistically reproduced the entirety of the observed pro-rich inequality in maternal CoC completion, falling to 78.7% when wealth was excluded. Given the observational, cross-sectional design, this indicates that observed inequality is closely associated with the selected socioeconomic and contextual circumstance variables, an interpretation broadly consistent with Roemer's Equality of Opportunity framework (41). This implies that reducing inequality will necessitate structural interventions alongside behavior-change strategies.

Across the three decomposition methods, women's and partner's education emerged as a relatively consistent contributor to wealth-related inequality, whereas radio exposure and perceived distance to a health facility contributed strongly in the Wagstaff decomposition but far less, and often non-significantly, in the Fairlie decomposition. Because the three methods decompose different aspects of inequality, the concentration index, the mean gap, and its non-linear counterpart, these results should be read as complementary rather than mutually confirming, and areas of divergence, such as those for radio exposure and perceived distance, are as informative as areas of convergence. This refines earlier Malawian evidence that identified geographical region as the largest single contributor (8), suggesting that regional disparities may partly reflect underlying differences in education, access to information, and practical barriers to care rather than geography *per se* (38, 40). The multilevel analysis further indicated that, although modest, cluster-level (PSU-level) variation in CoC completion persisted even after accounting for individual and household characteristics. This suggests an independent role for unmeasured contextual factors such as service availability, staffing, and local norms surrounding maternal healthcare. Collectively, these findings support strengthening community health worker programs and mobile outreach in underserved areas, expanding girls’ and women's education, and encouraging male involvement in maternal healthcare (24), particularly given that most of the observed wealth-related inequality in maternal CoC completion was statistically reproduced by circumstance variables such as wealth, residence, region, and partner's education.

### Policy implications

Based on the findings improving completion of the continuum of care in Malawi may require attention to both individual- and contextual-level barriers to maternal and child health service utilisation, in line with priorities already reflected in Malawi's Health Sector Strategic Plan. Given the strength and consistency of the association between women's education and CoC completion, continued investment in girls’ education and in demand-generation strategies that reach less-educated women, including community health worker outreach and mobile/outreach clinics in underserved areas, may be a productive entry point. The independent association of partner's education with completion, together with existing qualitative evidence on the benefits of male engagement in maternal healthcare, supports continued efforts to involve male partners in antenatal and postnatal care rather than treating maternal health as a woman-only concern. The persistent association between perceived distance and completion, alongside modest but persistent cluster-level variation, points to a continued role for improving geographic and financial access to services for women from poorer and rural households and for addressing local supply-side differences in service delivery. Reducing socioeconomic inequality in CoC completion is therefore likely to require a combination of demand-side interventions (education, male engagement, information and outreach) and supply-side and structural measures (facility access, service quality, and household-level poverty reduction) rather than either alone.

### Limitations

This study has several limitations. First, the analysis relied on self-reported DHS data, which may be subject to recall and social desirability bias. Second, the cross-sectional design precludes establishing causal relationships between the explanatory variables and continuum of care completion. Third, the DHS does not capture supply-side factors, such as health facility readiness and quality of care, which may influence completion of the continuum of care. Fourth, the DHS measure of perceived distance to a health facility is a subjective report that may conflate physical distance, transport availability, cost, and prior experience with the referral system; future work linking DHS data with GIS-based facility location data or Service Provision Assessment data would help disentangle these dimensions. Finally, although nationally representative data were used, the analyses were limited to variables available in the DHS, and unmeasured contextual factors may have influenced the observed associations.

## Data Availability

The datasets analyzed in this study (Malawi DHS 2010, 2015-16 and 2024) are publicly available from The DHS Program at https://dhsprogram.com/data/available-datasets.cfm?ctryid=24, following registration and approval of a data-use request.

## Conclusion

This study examined socioeconomic inequality in the completion of the eleven-component maternal, newborn, and child health (MNCH) continuum of care in Malawi using three rounds of nationally representative Demographic and Health Survey data. Although completion of the continuum of care improved over the study period, pro-rich socioeconomic inequality remained. Women's education, particularly higher education, was consistently associated with higher completion of both the maternal and full continuum of care, while partner's education and perceived distance to a health facility remained important correlates of completion. The findings further suggest that much of the observed wealth-related inequality in continuum of care completion is associated with socioeconomic and contextual circumstances, underscoring the persistent in8uence of structural disadvantage.
