# Supplementary material for: Socioeconomic inequality in the completion of the continuum of care for maternal, newborn, and child health in Malawi
Source: Front Reprod Health. 2026 Sep 30;8:1919232. doi: 10.3389/frph.2026.1919232 (PMC13661951; doi:10.3389/frph.2026.1919232)
Supplement: Supplementary file 1 [file Table1.docx]

**Data Sheet 1**

Supplementary Material

**Socioeconomic inequality in the completion of the continuum of care for maternal, newborn, and child health in Malawi**

*Redson Mwandama and Spy Munthali*

Frontiers in Reproductive Health

**Table S1.** Sensitivity analysis: continuum of care (CoC) under the ANC4-only definition versus the primary early-ANC4+ definition.

**Panel A. Prevalence by survey year (ANC4-only definition)**

| **Survey year** | **N** | **n completing** | **% completing** |
| --- | --- | --- | --- |
| ***Maternal CoC*** | | | |
| 2010 | 6,901 | 1,375 | 19.92 |
| 2015–16 | 5,211 | 1,287 | 24.70 |
| 2024 | 3,440 | 1,606 | 46.69 |
| Pooled | 15,552 | 4,268 | 27.44 |
| ***Full CoC*** | | | |
| 2010 | 3,442 | 247 | 7.18 |
| 2015–16 | 2,523 | 238 | 9.43 |
| 2024 | 1,660 | 293 | 17.65 |
| Pooled | 7,625 | 778 | 10.20 |

**Panel B. Erreygers concentration index, pooled**

| **Outcome / definition** | **N** | **Erreygers CI (95% CI)** | **p-value** |
| --- | --- | --- | --- |
| Maternal CoC — primary (early ANC4+) | 15,552 | 0.0357 (0.0209–0.0504) | <0.001 |
| Maternal CoC — ANC4-only | 15,552 | 0.1158 (0.0936–0.1379) | <0.001 |
| Full CoC — primary (early ANC4+) | 7,625 | 0.0038 (−0.0072–0.0149) | 0.497 |
| Full CoC — ANC4-only | 7,625 | 0.0300 (0.0103–0.0498) | 0.003 |

**Panel C. Cross-classification of the two definitions**

*Maternal CoC (N = 15,552)*

|  | **ANC4-only = 0** | **ANC4-only = 1** | **Total** |
| --- | --- | --- | --- |
| Primary = 0 | 11,284 | 2,765 | 14,049 |
| Primary = 1 | 0 | 1,503 | 1,503 |
| Total | 11,284 | 4,268 | 15,552 |

*Full CoC (N = 7,625)*

|  | **ANC4-only = 0** | **ANC4-only = 1** | **Total** |
| --- | --- | --- | --- |
| Primary = 0 | 6,847 | 496 | 7,343 |
| Primary = 1 | 0 | 282 | 282 |
| Total | 6,847 | 778 | 7,625 |

*Note: Definitions are nested (every primary completer is also an ANC4-only completer). Counts and percentages in this table are unweighted. Under early ANC4+, unweighted maternal CoC prevalence is 9.7% and full CoC is 3.7%, versus 27.4% and 10.2% under ANC4-only (survey-weighted estimates for the primary definition are given in Table 2 of the main text).*

**Table S2.** Sensitivity analysis: adjusted odds ratios under the ANC4-only definition versus the primary early-ANC4+ definition (pooled).

**Panel A. Maternal CoC**

| **Variable / category** | **Primary (early ANC4+)** | **ANC4-only** |
| --- | --- | --- |
| ***Wealth quintile*** | | |
| Poorest (ref.) | 1.00 | 1.00 |
| Poorer | 0.97 (0.76–1.22) | 1.11 (0.96–1.29) |
| Middle | 0.94 (0.74–1.18) | 1.20 (1.03–1.39)* |
| Richer | 1.16 (0.92–1.46) | 1.33 (1.14–1.55)*** |
| Richest | 0.97 (0.72–1.29) | 1.24 (1.01–1.51)* |
| ***Woman's education*** | | |
| No education (ref.) | 1.00 | 1.00 |
| Primary | 1.23 (0.94–1.60) | 1.08 (0.91–1.30) |
| Secondary | 1.50 (1.08–2.08)* | 1.27 (1.01–1.60)* |
| Higher | 3.42 (1.98–5.90)*** | 2.32 (1.47–3.65)*** |
| ***Partner's education*** | | |
| No education (ref.) | 1.00 | 1.00 |
| Primary | 1.22 (0.90–1.66) | 1.08 (0.89–1.31) |
| Secondary | 1.14 (0.82–1.59) | 1.16 (0.94–1.42) |
| Higher | 1.59 (0.98–2.59) | 1.70 (1.22–2.39)** |
| ***Residence / distance*** | | |
| Rural (vs urban) | 1.01 (0.77–1.34) | 0.89 (0.75–1.05) |
| Distance a big problem | 0.83 (0.72–0.96)* | 0.86 (0.78–0.95)** |
| ***Media exposure*** | | |
| Radio (yes) | 1.14 (0.96–1.34) | 1.15 (1.03–1.28)** |
| Newspaper (yes) | 0.92 (0.75–1.12) | 1.16 (1.03–1.32)* |
| TV (yes) | 1.14 (0.94–1.40) | 1.06 (0.93–1.21) |
| ***Age / parity*** | | |
| Age (years) | 1.02 (1.00–1.05)* | 1.02 (1.01–1.04)** |
| Parity | 0.91 (0.84–0.97)** | 0.91 (0.87–0.96)*** |
| ***Region*** | | |
| Central (vs Northern) | 0.75 (0.62–0.92)** | 0.82 (0.72–0.95)** |
| Southern (vs Northern) | 0.76 (0.63–0.92)** | 0.71 (0.62–0.81)*** |
| ***Survey year*** | | |
| 2015–16 (vs 2010) | 1.93 (1.54–2.42)*** | 1.16 (1.02–1.32)* |
| 2024 (vs 2010) | 3.64 (2.88–4.58)*** | 2.69 (2.36–3.07)*** |
| N | 15,552 | 15,552 |

**Panel B. Full CoC**

| **Variable / category** | **Primary (early ANC4+)** | **ANC4-only** |
| --- | --- | --- |
| ***Wealth quintile*** | | |
| Poorest (ref.) | 1.00 | 1.00 |
| Poorer | 0.74 (0.46–1.18) | 1.14 (0.86–1.52) |
| Middle | 0.78 (0.49–1.25) | 1.08 (0.81–1.45) |
| Richer | 0.86 (0.52–1.40) | 1.08 (0.80–1.46) |
| Richest | 0.72 (0.41–1.28) | 1.27 (0.83–1.95) |
| ***Woman's education*** | | |
| No education (ref.) | 1.00 | 1.00 |
| Primary | 2.10 (1.14–3.84)* | 1.23 (0.87–1.73) |
| Secondary | 2.64 (1.28–5.42)** | 1.52 (0.99–2.32) |
| Higher | 3.45 (1.04–11.4)* | 1.96 (0.79–4.88) |
| ***Partner's education*** | | |
| No education (ref.) | 1.00 | 1.00 |
| Primary | 0.78 (0.43–1.40) | 1.16 (0.81–1.65) |
| Secondary | 1.25 (0.66–2.34) | 1.38 (0.93–2.05) |
| Higher | 1.79 (0.76–4.23) | 1.18 (0.64–2.20) |
| ***Residence / distance*** | | |
| Rural (vs urban) | 1.40 (0.84–2.35) | 1.19 (0.84–1.69) |
| Distance a big problem | 0.85 (0.63–1.15) | 0.87 (0.72–1.06) |
| ***Media exposure*** | | |
| Radio (yes) | 1.13 (0.84–1.52) | 1.24 (1.01–1.52)* |
| Newspaper (yes) | 0.99 (0.66–1.46) | 1.00 (0.79–1.27) |
| TV (yes) | 0.73 (0.50–1.05) | 0.97 (0.74–1.28) |
| ***Age / parity*** | | |
| Age (years) | 0.99 (0.96–1.03) | 0.99 (0.96–1.02) |
| Parity | 1.03 (0.90–1.19) | 0.99 (0.90–1.08) |
| ***Region*** | | |
| Central (vs Northern) | 0.71 (0.48–1.03) | 0.99 (0.75–1.33) |
| Southern (vs Northern) | 0.65 (0.45–0.96)* | 0.98 (0.73–1.30) |
| ***Survey year*** | | |
| 2015–16 (vs 2010) | 2.59 (1.71–3.92)*** | 1.30 (1.01–1.68)* |
| 2024 (vs 2010) | 4.69 (3.05–7.20)*** | 2.61 (2.03–3.36)*** |
| N | 7,625 | 7,625 |

*Note: Survey-weighted logistic regression (svy: logit) with the full covariate set and survey-round fixed effects. Primary columns in Panel A reproduce the pooled maternal CoC estimates in Table 7 of the main text; the primary full CoC column in Panel B is reported here and underlies the pooled full CoC adjusted odds ratio cited in the Results. * p < 0.05; ** p < 0.01; *** p < 0.001.*

**Table S3.** Sensitivity analysis: 2024 ANC8+ and early-ANC8+ definitions versus the primary early-ANC4+ definition (2024 only).

**Panel A. Prevalence, 2024**

| **Definition** | **Outcome** | **n** | **%** |
| --- | --- | --- | --- |
| ANC8+ (≥8 visits, no timing) | Maternal CoC | 75 | 2.18 |
| Early ANC8+ (≥8 visits + early) | Maternal CoC | 58 | 1.69 |
| ANC8+ (≥8 visits, no timing) | Full CoC | 14 | 0.84 |
| Early ANC8+ (≥8 visits + early) | Full CoC | 13 | 0.78 |

*Note: Maternal CoC N = 3,440; full CoC N = 1,660.*

**Panel B. Erreygers concentration index, 2024**

| **Outcome / definition (2024)** | **N** | **Erreygers CI (95% CI)** | **p-value** |
| --- | --- | --- | --- |
| Maternal CoC — primary (early ANC4+) | 3,440 | 0.0606 (0.0257–0.0956) | <0.001 |
| Maternal CoC — ANC8+ | 3,440 | 0.0230 (0.0091–0.0368) | 0.001 |
| Maternal CoC — early ANC8+ | 3,440 | 0.0221 (0.0090–0.0352) | 0.001 |
| Full CoC — primary (early ANC4+) | 1,660 | 0.0105 (−0.0189–0.0398) | 0.485 |
| Full CoC — ANC8+ | 1,660 | 0.0053 (−0.0082–0.0188) | 0.440 |
| Full CoC — early ANC8+ | 1,660 | 0.0040 (−0.0093–0.0172) | 0.558 |

**Panel C. Adjusted odds ratios, 2024 (exploratory)**

*Maternal CoC, 2024 (N = 3,440)*

| **Variable / category** | **Primary** | **ANC8+** | **Early ANC8+** |
| --- | --- | --- | --- |
| ***Wealth quintile*** | | | |
| Poorer (vs poorest) | 1.13 (0.78–1.64) | 0.46 (0.13–1.58) | 0.14 (0.02–0.89)* |
| Middle | 0.99 (0.70–1.40) | 1.22 (0.45–3.31) | 1.45 (0.50–4.17) |
| Richer | 1.23 (0.83–1.81) | 1.81 (0.63–5.23) | 1.96 (0.61–6.34) |
| Richest | 1.15 (0.76–1.75) | 1.14 (0.44–2.94) | 1.05 (0.35–3.13) |
| ***Woman's education*** | | | |
| Primary (vs none) | 1.15 (0.69–1.92) | 1.04 (0.15–7.21) | 0.81 (0.10–6.38) |
| Secondary | 1.46 (0.81–2.63) | 3.36 (0.43–26.3) | 3.45 (0.38–31.6) |
| Higher | 2.77 (1.17–6.58)* | 6.22 (0.57–67.4) | 6.83 (0.53–88.1) |
| ***Partner's education*** | | | |
| Primary (vs none) | 0.94 (0.58–1.54) | 0.49 (0.12–1.95) | 0.57 (0.11–3.00) |
| Secondary | 0.86 (0.52–1.42) | 0.18 (0.04–0.74)* | 0.21 (0.04–1.14) |
| Higher | 1.04 (0.52–2.06) | 0.43 (0.10–1.93) | 0.56 (0.10–3.06) |
| ***Other covariates*** | | | |
| Rural (vs urban) | 1.18 (0.85–1.64) | 0.39 (0.21–0.72)** | 0.40 (0.20–0.80)** |
| Distance a big problem | 0.79 (0.64–0.98)* | 0.90 (0.51–1.61) | 0.86 (0.45–1.64) |
| Radio (yes) | 1.07 (0.84–1.37) | 0.63 (0.29–1.36) | 0.53 (0.22–1.25) |
| Newspaper (yes) | 1.14 (0.86–1.52) | 1.55 (0.77–3.11) | 1.45 (0.67–3.10) |
| TV (yes) | 1.22 (0.92–1.64) | 0.80 (0.41–1.57) | 0.98 (0.46–2.10) |
| Age (years) | 1.01 (0.98–1.04) | 1.02 (0.93–1.11) | 1.00 (0.92–1.10) |
| Parity | 1.01 (0.91–1.11) | 0.95 (0.64–1.43) | 1.05 (0.69–1.58) |
| Central (vs Northern) | 0.70 (0.52–0.94)* | 0.69 (0.35–1.38) | 1.05 (0.48–2.29) |
| Southern (vs Northern) | 0.71 (0.53–0.96)* | 0.36 (0.17–0.73)** | 0.47 (0.21–1.07) |

*Full CoC, 2024 (primary N = 1,660; ANC8+ and early-ANC8+ effective N = 1,557 )*

| **Variable / category** | **Primary** | **ANC8+** | **Early ANC8+** |
| --- | --- | --- | --- |
| ***Wealth quintile*** | | | |
| Poorer (vs poorest) | 0.92 (0.46–1.84) | 0.33 (0.03–3.29) | 0.33 (0.03–3.36) |
| Middle | 0.99 (0.48–2.04) | 2.70 (0.57–12.7) | 2.86 (0.61–13.4) |
| Richer | 0.81 (0.36–1.81) | 1.12 (0.16–7.85) | 1.22 (0.17–8.63) |
| Richest | 1.25 (0.57–2.72) | 1.30 (0.22–7.75) | 1.12 (0.16–7.92) |
| ***Woman's education*** | | | |
| Primary (vs none) | 1.19 (0.50–2.85) | 0.12 (0.00–8.16) | 0.12 (0.00–9.22) |
| Secondary | 2.62 (0.89–7.69) | 0.57 (0.01–31.8) | 0.53 (0.01–34.0) |
| Higher | 1.79 (0.28–11.4) | — | — |
| ***Partner's education*** | | | |
| Primary (vs none) | 0.25 (0.11–0.56)*** | 0.09 (0.02–0.39)** | 0.09 (0.02–0.40)** |
| Secondary | 0.39 (0.16–0.94)* | 0.06 (0.01–0.29)*** | 0.06 (0.01–0.29)*** |
| Higher | 0.59 (0.16–2.24) | 0.06 (0.00–1.71) | 0.07 (0.00–2.14) |
| ***Other covariates*** | | | |
| Rural (vs urban) | 1.80 (0.87–3.72) | 0.46 (0.10–2.15) | 0.52 (0.10–2.74) |
| Distance a big problem | 0.77 (0.49–1.20) | 0.76 (0.28–2.07) | 0.82 (0.30–2.24) |
| Radio (yes) | 1.13 (0.72–1.76) | 0.95 (0.31–2.90) | 1.08 (0.35–3.35) |
| Newspaper (yes) | 1.14 (0.59–2.19) | 2.23 (0.77–6.49) | 1.77 (0.62–5.05) |
| TV (yes) | 0.54 (0.32–0.94)* | 0.37 (0.10–1.38) | 0.44 (0.11–1.70) |
| Age (years) | 0.96 (0.91–1.01) | 0.88 (0.81–0.96)** | 0.89 (0.82–0.97)** |
| Parity | 1.24 (1.02–1.51)* | 1.83 (1.28–2.60)*** | 1.78 (1.27–2.49)*** |
| Central (vs Northern) | 0.76 (0.45–1.27) | 0.46 (0.14–1.50) | 0.54 (0.15–1.86) |
| Southern (vs Northern) | 0.55 (0.32–0.96)* | 0.17 (0.04–0.75)* | 0.19 (0.04–0.90)* |

*Note: Survey-weighted logistic regression. Primary columns reproduce the 2024 estimates in Table 7 of the main text (maternal CoC) and the 2024 full CoC model. ANC8+ and early-ANC8+ outcomes are rare (13–75 events); estimates should be treated as exploratory. In the full-CoC ANC8+ models, the “no education” and “higher” woman’s-education categories were dropped because of perfect prediction (effective N = 1,557). — = not estimable. * p < 0.05; ** p < 0.01; *** p < 0.001.*
